# The combined self- and parent-rated SDQ score profile predicts care use and psychiatric diagnoses

**DOI:** 10.1007/s00787-020-01667-5

**Published:** 2020-10-30

**Authors:** Jorien Vugteveen, Annelies de Bildt, Catharina A. Hartman, Sijmen A. Reijneveld, Marieke E. Timmerman

**Affiliations:** 1 Heymans Institute for Psychological Research, University of Groningen, Groningen, The Netherlands; 2 Department of Psychiatry, Child and Adolescent Psychiatry, University Medical Center Groningen (UMCG), University of Groningen, Groningen, The Netherlands; 3 Accare Child and Adolescent Psychiatry, Groningen, The Netherlands; 4 Department of Psychiatry, Interdisciplinary Center Psychopathology and Emotion Regulation (ICPE), University Medical Center Groningen (UMCG), University of Groningen, Groningen, The Netherlands; 5 Department of Health Sciences, University Medical Center Groningen (UMCG), University of Groningen, Groningen, The Netherlands; 6 Netherlands Organisation of Applied Scientific Research (TNO), Leiden, The Netherlands

**Keywords:** Adolescent mental health, Comorbidity, Multi-informant, Three-step multilevel mixture modelling

## Abstract

The Strengths and Difficulties Questionnaire (SDQ) is widely used, based on evidence of its value for screening. This evidence primarily regards the single informant total difficulties scale and separate difficulties subscales. We assessed to what degree adolescents’ SDQ profiles that combined all self- and parent-rated subscales were associated with use of care and psychiatric diagnoses, and examined the added value thereof over using only a single informant and the total scale. Cluster analysis was used to identify common SDQ profiles based on self- and parent-reports among adolescents aged 12–17 in mental healthcare (*n* = 4282), social care (*n* = 124), and the general population (*n* = 1293). We investigated associations of the profiles with ‘care use’ and ‘DSM-IV diagnoses’, depending on gender. We identified six common SDQ profiles: five profiles with varying types and severities of reported difficulties, pertaining to 95% of adolescents in care, and one without difficulties, pertaining to 55% of adolescents not in care. The types of reported difficulties in the profiles matched DSM-IV diagnoses for 88% of the diagnosed adolescents. The SDQ profiles were found to be more useful for predicting care use and diagnoses than SDQ scores reported by the adolescent as single informant and the total difficulties scale. The latter indicated the presence of problems among 42–63% of the adolescents in care, missing a substantial number of adolescents with reported emotional difficulties and borderline problem severity. These findings advocate the use of combined self- and parent-rated SDQ score profiles for screening.

**Electronic supplementary material:**

The online version of this article (10.1007/s00787-020-01667-5) contains supplementary material, which is available to authorized users.

## Introduction

Approximately 15–25% of adolescents experience psychiatric problems [1, 2]. To receive adequate mental healthcare, these problems need to be effectively detected and diagnosed. To that end, it is recommended that clinicians consider information on the adolescent’s psychosocial functioning provided by multiple informants [3], for instance the adolescents themselves and their parents. Ratings from multiple informants are considered complementary, with more informants better reflecting differences in perspective [4–6]. One way to gather multiple-informant information for the purpose of screening for psychosocial problems is to ask the informants to complete a questionnaire, such as the widely used Strengths and Difficulties Questionnaire (SDQ) [7, 8]. The SDQ contains five subscales (four related to psychosocial difficulties, and one to strengths) and one total difficulties scale.

The validity of the adolescent self- and parent-rated SDQ versions for screening is typically investigated by assessing their usefulness for two purposes. The first is distinguishing between adolescents from general and mental healthcare populations, for which the self-rated [9–11] and the parent-rated [10] total difficulties scales are considered sufficiently useful. The second purpose is predicting the presence of specific disorders regarded to be content-wise related to the constructs measured by the SDQ [12, 13] among adolescents from mental healthcare populations. Parent ratings were consistently found to be useful for predicting Attention-Deficit/Hyperactivity Disorder (ADHD), Conduct/Oppositional Defiant Disorder (CD/ODD) [14–16], and Autism Spectrum Disorder (ASD) [16]. Findings regarding adolescent ratings varied substantially, some supporting their usefulness for predicting ADHD and CD/ODD [15, 16], but not for ASD [16]. For Anxiety/Mood disorder, findings on the adolescent and parent ratings were too diverse for meaningful conclusions [14–16]. Besides, most studies focused on either the adolescent or the parent as informant, therewith providing limited information to inform clinical practice about the usefulness of the recommended multi-informant ratings for screening. Evidence on the latter is lacking.

An additional peculiarity shared by the available studies described above is that they provide information about single domains of behavior measured by the SDQ (one of the four difficulties scales) or about an adolescent’s problem behavior in general (i.e., total difficulty scale, without distinguishing between the domains) and not on the value of using multi-domain SDQ information for screening. One weakness of this approach is that considering only the total difficulties scale for distinguishing between the general and mental healthcare populations potentially results in clinicians overlooking groups of adolescents experiencing a single type of problems, as they may not score particularly high on the total difficulties scale. Another weakness of considering the total difficulties scale or the separate difficulties subscales for predicting specific disorders is that it provides limited information about the potential presence of co-occurring disorders. That is, the outcome criterion in studies considering the total difficulties scale was typically the presence of at least one disorder, regardless of their total number and specific type(s). The outcome criterion in studies considering separate difficulty subscales was the presence of one specific type of disorder per subscale. With high comorbidity rates in youth with psychiatric problems [17], this approach over-simplifies reality, with the consequence that the findings from these studies have needlessly limited relevance for clinicians.

The aim of this study is to surpass the limitations of existing findings by assessing whether using adolescents’ SDQ profiles that combine all self- and parent-rated SDQ subscales, have added value over using a single informant and the total scale in predicting use of care and psychiatric diagnoses. We will do so by first identifying common SDQ profiles based on self- and parent-reports among adolescents aged 12–17 in child and adolescent mental healthcare (CAMH), child and adolescent social care (CASC), and the general population (community setting). We selected these populations because they represent populations with relatively many adolescents with one or more psychiatric disorders (CAMH), with various psychosocial problems (CASC), and little to no psychiatric problems (community setting) [18]. Next, we will investigate associations of these SDQ profiles with ‘care use’ and ‘DSM-IV diagnoses’ (i.e., ADHD, CD/ODD, Anxiety/Mood, ASD, including co-occurring disorders) among diagnosed adolescents, depending on gender. Exploring the potential presence of a gender effect on the usefulness of SDQ profiles for screening can provide further insight as to how to optimize the use of these profiles in clinical practice.

## Methods

### Samples

Data were collected from 5699 12- to 17-year-old Dutch adolescents and their parents. These adolescents were referred to care (CAMH and CASC settings) or were part of the general population (community setting).

#### CAMH setting

Data were collected as part of routine outcome monitoring during intake at two large mental healthcare providers in the Netherlands: between 2011 and 2013 data were collected from 229 adolescents referred to a mental healthcare provider and between 2013 and 2015 from 4053 adolescents referred to another mental healthcare provider. For the 4282 adolescents in this sample, adolescent-reported SDQ data (*n* = 367), parent-reported SDQ data (*n* = 245) or both (*n* = 3670) were available. In this sample, 2915 adolescents received a DSM-IV diagnosis [3] in any of the four categories (Anxiety/Mood disorder, ASD, CD/ODD, and ADHD) that content-wise respond to the SDQ subscales (see Table 1). The diagnoses were established by trained psychologists/psychiatrists in a multidisciplinary team. Another 635 adolescents were diagnosed with other DSM-IV diagnoses and 732 had no registered diagnosis, because they did not meet the DSM-IV criteria for any disorder.

**Table 1:** Prevalence and comorbidity with other disorders per DSM-IV diagnosis category among 2915 diagnosed adolescents within the CAMH sample

DSM category	Gender	Single diagnosis	Comorbid with …				Total
			Anxiety/Mood	CD/ODD	ADHD	ASD	
Anxiety/Mood	All^b^	1152	–	26	103	111	1392
	M	297	–	12	38	51	398
	F	851	–	14	63	60	988
CD/ODD	All^b^	195	26	–	138	11	370
	M	128	12	–	106	8	254
	F	63	14	–	30	3	110
ADHD	All^b^	537	103	138	–	110	888
	M	361	38	106	–	89	594
	F	174	63	30	–	21	288
ASD	All^b^	486	111	11	110	–	718
	M	313	51	8	89	–	416
	F	167	60	3	21	–	251
Multi-problem^a^	All^b^						46
	M						35
	F						10

*Anxiety/Mood *anxiety/mood disorder, *ASD *autism spectrum disorder, *CD/ODD *conduct/oppositional defiant disorder, *ADHD *attention-deficit/hyperactivity disorder, *M *male adolescents, *F *female adolescents

^a^Adolescents diagnosed with three or more of the above mentioned disorders

^b^Note that the number of male and female adolescents may not add up to the total number of adolescents because information on gender is missing for 58 adolescents in the CAMH sample

#### CASC setting

The CASC data pertains to 124 12- to 17-year-olds referred to four large child and adolescent social care providers in the Netherlands, from whom adolescent-reported SDQ data (*n* = 19), parent-reported SDQ data (*n* = 31) or both (*n* = 74) were collected between 2011 and 2013.

#### Community setting

The data were collected at schools for secondary and intermediate vocational education spread throughout the Netherlands in three waves: (1) in 2009/2010 data were collected from 519 13- to 14-year-old adolescents, (2) between 2011 and 2013 from 331 12- to 17-year-olds, and 3) in 2016/2017 from 443 similarly aged adolescents. For these 1293 adolescents, adolescent-reported SDQ data (*n* = 452), parent-reported SDQ data (*n* = 69) or both (*n* = 772) were available. A substantial part of the data from the first wave was collected as part of a scheduled well-child assessment.

Table 2 provides demographic information on the three samples of adolescents and, for comparison, on the Dutch population [19]. The information in the table shows that adolescents with an ‘other than Dutch’ ethnic background and 12- and 17-year-olds are somewhat underrepresented in the sample from the community setting. For the sample from the CASC setting and for a small part of the sample from the CAMH setting (i.e., the 229 participants from whom data were collected between 2011 and 2013), there are indications that they are representative for the populations that they were drawn from [20]. For the rest of the sample from the CAMH setting we cannot provide an indication of representativity.

**Table 2:** Demographic characteristics of the adolescents in the community, CASC and CAMH samples

Characteristics	Community (*n* = 1293)	CASC (*n* = 124)	CAMH (*n* = 4282)	Dutch population
	*N* (%^a^)	*N* (%)	*N* (%)	%
Gender				
Male	623 (48.4)^b^	48 (38.7)	2006 (47.5)^c^	49.5
Female	664 (51.6)	76 (61.3)	2218 (52.5)	50.5
Age				
12	99 (7.7)^d^	9 (7.3)	615 (14.4)	16.5
13	354 (27.6)	19 (15.3)	785 (18.3)	16.3
14	336 (26.2)	20 (16.1)	816 (19.1)	16.4
15	191 (14.9)	24 (19.4)	838 (19.6)	16.9
16	178 (13.9)	30 (24.2)	713 (16.7)	16.9
17	126 (9.8)	22 (17.7)	515 (12.0)	17.1
Native country mother				
The Netherlands	1045 (86.2)^e^	92 (88.5)^f^	201 (94.4)^g^	78.6
Other	168 (13.8)	12 (11.5)	12 (5.6)	21.4
Educational level mother				
Low	258 (24.3)^h^	43 (43.9)^i^	59 (28.1)^j^	23.6
Medium	439 (41.3)	50 (51.0)	109 (51.9)	41.7
High	365 (34.4)	5 (5.1)	42 (20.0)	34.7

*CASC *child and adolescent social care, *CAMH* child and adolescent mental health

^a^Percentages computed of valid cases only

^b^Missing: *n* = 6

^c^Missing: *n* = 58

^d^Missing: *n* = 9, exact age unknown, but definitely between 12 and 17 years old

^e^Missing: *n* = 80

^f^Missing: *n* = 20

^g^Missing: *n* = 4069

^h^Missing: *n* = 231

^i^Missing: *n* = 26

^j^Missing: *n* = 4072

### The Strengths and Difficulties Questionnaire

The 25 items of the Dutch adolescent- and parent-reported versions of the SDQ are evenly divided over five subscales: one for strengths (prosocial behavior) and four subscales difficulties (emotional, conduct, hyperactivity, and social problems) [7, 8, 21]. The total difficulties scale consists of the summed four difficulties subscale scores. Additionally, the SDQ contains an impact scale that aims to measure the impact (i.e., the chronicity, distress, social impairment for the adolescent, and burden for others) of the reported difficulties among adolescents. All items are rated on a three-point scale (0 = not true, 1 = somewhat true and 2 = certainly true). Five positively worded items belonging to different difficulties subscales are reverse-coded. High scores on the four difficulties subscales and the total difficulties scale, represent a high degree of difficulties; a high score on the prosocial subscale represents a high degree of prosocial behavior. A high score on the impact scale represents a high degree of impact. Table S1 (available online) reports mean scale scores and standard deviations per setting (community, CASC, CAMH) and informant (adolescent, parent). The information shows that within the community setting adolescents reported higher severity for most types of difficulties than their parents did, and weaker prosocial skills. Within the CAMH setting, the opposite was found. The findings regarding both settings are in line with previous research [15, 21]. Within the CASC setting, adolescents reported lower conduct problem severity than their parents did. Additionally, parents within the CASC and CAMH settings reported higher impact of psychosocial difficulties than the adolescents did. No informant differences were found for the remaining subscales.

### Statistical analysis

We assessed the degree to which adolescents’ SDQ profiles were associated with use of care and psychiatric diagnoses by performing a three-step multilevel mixture analysis [22] in LatentGold [23] on all available adolescent self- and parent-rated SDQ difficulties subscales simultaneously, thus assuming the data missing at the informant level as missing at random. The impact scale was not included in the analyses, as these scores are not meaningful in themselves: Impact scale scores can only be meaningfully interpreted in combination with difficulties scale scores, with the complicating issue that the meaning of impact scale scores differs across difficulties scales (see for example the SDQ scoring algorithm on the SDQ website: https://www.sdqinfo.org/c4.html). This prevents a straightforward meaningful inclusion of this scale in estimating the SDQ profiles. The first step in the analysis was to identify clusters of adolescents with common SDQ profiles by estimating multilevel mixture models containing one to eight clusters, all with the five SDQ subscales as ordinal dependent variables, the informant (self, parent) at level 1, and the adolescent at level 2. The model with the smallest Bayes Information Criterion (BIC) [24] value was selected for further analysis. The SDQ profiles found were interpreted using British cutoff scores to classify their adolescent self- and parent-reported mean SDQ scale scores as ‘normal’, ‘borderline’, or ‘abnormal’ [7, 9]. Informant differences were tested using paired sample *t* tests, with *α* = 0.01 and Bonferroni correction for multiple comparisons per cluster. The second step in the analysis was to retrieve the posterior cluster membership probabilities for the selected model. The third and final step was to relate the SDQ profiles to (1) ‘care use’, by relating cluster membership to setting (community, CASC, and CAMH) and (2) ‘DSM diagnoses’ for adolescents receiving CAMH, by relating cluster membership to type of diagnosis (Anxiety/Mood disorder, CD/ODD, ADHD, ASD, and combinations). For both, the interaction with gender was also included. For illustration purposes, perturbed data and example code for both steps of the analysis are available on https://osf.io/87h45/. Additionally, we provide code that can be used to estimate an individual’s probabilities for displaying each of the profiles identified in this study based on any combination of observed SDQ subscale scores. In other words, this syntax can be used to score new cases. Note that the resulting probabilities bear some uncertainty, because the samples used in this study are not random samples from their respective populations. For that same reason, and because the prevalence of disorders in the population of adolescents is unknown to us, we cannot provide a syntax for estimating the individual’s probabilities for each (combination) of the disorders.

The SDQ is considered potentially useful for predicting use of care if (a) the SDQ profiles indicating the absence of psychiatric problems are mainly prevalent among adolescents not in care and (b) the SDQ profiles indicating presence of psychiatric problems are mainly prevalent among adolescents in care, especially those from the CAMH setting. The SDQ is considered useful for obtaining preliminary indications of the disorders present among adolescents if the reported difficulties in the SDQ profiles match the diagnosed disorders.

For conciseness, only gender differences in profile prevalence estimates ≥ 20% are reported in Tables 3 and 4. The remaining gender differences can be found in Tables S2 and S3 (available online). Prevalence estimates are not reported for (combinations of) disorders that fewer than 100 adolescents within our CAMH sample were diagnosed with.

**Table 3:** Per setting, SDQ profile prevalence estimates in percentages

Setting	SDQ profile					
	No difficulties	Borderline hyperactivity difficulties	Borderline conduct and social difficulties	Emotional difficulties	Emotional and social difficulties	Overall difficulties
	% All (M/F)^a^	% All (M/F)^a^	% All (M/F)^a^	% All (M/F)^a^	% All (M/F)^a^	% All (M/F)^a^
Community	55	15	17	9	4	1
In care (total)	5	18	16	20 (8/32)	21 (11/32)	20
CASC	2	18 (4/27)	34 (57/20)	8	7	31
CAMH	5	18	16	20 (8/32)	22 (11/32)	20

*SDQ *Strengths and Difficulties Questionnaire, *CASC *Child and Adolescent Social Care, *CAMH* Child and Adolescent Mental Healthcare

^a^Profile prevalence estimates in percentages for males and females are reported for gender differences > 20%

**Table 4:** SDQ profile prevalence estimates in percentages per (combination of) disorder(s) diagnosed among adolescents using child and adolescent mental healthcare (CAMH)

DSM-IV diagnosis	SDQ profile					
	No difficulties	Borderline hyperactivity difficulties	Borderline conduct and social difficulties	Emotional difficulties	Emotional and social difficulties	Overall difficulties
	% All (M/F)^a^	% All (M/F)^a^	% All (M/F)^a^	% All (M/F)^a^	% All (M/F)^a^	% All (M/F)^a^
Anxiety/Mood	3	5	6	**39**	**38**	**9**
CD/ODD	4	22	**35**	2	3	**33**
ADHD	3	**57 (65/41)**	2	4	6	**29**
ASD	2	1	**42 (50/26)**	7	**28**	**21**
Anxiety/Mood and ADHD	1	**20 (40/8)**	0	**20**	**32**	**26**
Anxiety/Mood and ASD	0	0	**7**	**17**	**66 (50/80)**	**10**
CD/ODD and ADHD	0	**36**	**6**	0	0	**58**
ADHD and ASD	2	**10**	**21 (26/01)**	0	**17**	**50 (44/75)**
Other^b^	10	15	13	36 (16/45)	16	10

Per disorder (combination), the percentages for content-wise matching SDQ profiles are printed in bold

*SDQ *Strengths and Difficulties Questionnaire, *M *male adolescents, *F *female adolescents, *CD/ODD *conduct disorder/oppositional defiant disorder, *ADHD *attention deficit disorder, *ASD* autism spectrum disorder

^a^Profile prevalence estimates for males and females are reported for gender differences > 0.20

^b^Adolescents diagnosed with DSM-IV disorders other than ADHD, CD/ODD, Anxiety/Mood disorder, ASD

Additional information on the usefulness of the SDQ score profile approach is provided in two ways. First, we compared the profile approach to the use of the total difficulties scale. Per setting (community, CASC, CAMH) and SDQ version, this was done by assessing profile prevalence estimates among adolescents with total difficulties scale scores within the ‘normal’ range and within the ‘borderline’/’abnormal’ range. Second, the profile approach was compared to the SDQ scoring algorithm that is available on the SDQ website. The profile approach and the algorithm approach were compared by assessing how well their outcomes matched the diagnosed disorders among the adolescents in the CAMH setting. The scoring algorithm resulted in ‘unlikely’, ‘possible’ or ‘probable’ predictions for emotional disorders, conduct disorders, hyperactivity disorders, or any of these disorders. We considered both ‘possible’ and ‘probable’ predictions to be indicative of the potential presence of a disorder. The profile approach resulted in the estimated prevalence of profiles per diagnosis group. The content of these profiles could either match the diagnosed disorder or not (e.g., a profile indicating only the presence of emotional difficulties matches Anxiety/Mood disorder, but not CD/ODD).

## Results

### Identifying common SDQ profiles

**Fig. 1:**
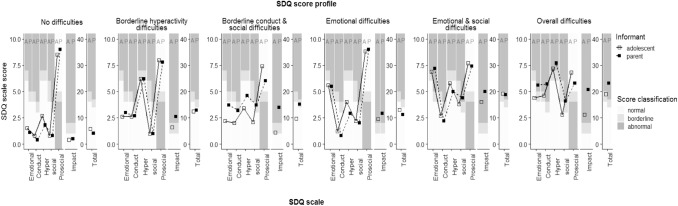
Adolescent self- (A) and parent-reported (P) mean scale scores per SDQ profile. Table S4 (available online) contains the numerical values of the scale scores presented here

Six clusters (i.e. groups) of adolescents, thus six common SDQ profiles, were identified. Per profile, Fig. 1 presents adolescent self- and parent-reported mean scale scores for the strengths and difficulties subscales and total difficulties scale, and their classification according to the range in which they fell (normal, borderline, abnormal). One group had a profile with means within the ‘normal’ range, thus we labelled it the ‘no difficulties’ profile. Two groups each had a profile with one or two mean subscale scores in the ‘borderline’ range. We labelled those the ‘borderline hyperactivity difficulties’ and ‘borderline conduct and social difficulties’ profiles, based on their affected domains. The remaining three groups each showed a profile containing one or more means in the ‘abnormal’ range. Based on their affected domains, we labelled them the ‘emotional difficulties’, ‘emotional and social difficulties’, and ‘overall difficulties’ profiles. Additionally, Fig. 1 shows that higher impact scale scores were generally reported for adolescents with SDQ score profiles that represented more severe and complex difficulties, with the parents reporting generally higher impact severity than the adolescents did.

#### Estimating an adolescent’s profile probabilities

The estimated multilevel mixture model can be applied to obtain an individual adolescent’s probability for each of the six profiles (code for scoring new cases is available on https://osf.io/87h45/). For illustrative purposes, we provide two examples here. First, for an adolescent with a maximum score (i.e., 10) on the adolescent-reported and parent-reported emotional difficulties scale, combined with scores of 7 on the remaining scales of both informant versions, the model results in an estimated probability of 0.99 for the ‘overall difficulties’ profile and 0.01 for the ‘emotional and social difficulties’ profile. Second, for an adolescent with a score of 5 on all adolescent-reported scales and a score of 4 on all parent-reported scales, the estimated probabilities are 0.51 for the ‘overall difficulties’ profile, 0.36 for the ‘emotional and social difficulties’ profile, and 0.13 for the ‘borderline conduct and social difficulties’ profile. To validate the stability of the 6-cluster solution across populations the cluster analysis was performed on the community data and on the CAMH data separately. The resulting profiles (Tables S5 and S6, available online) highly resembled the six profiles found in the combined samples.

### Identifying adolescents in need of care

Per setting (community, CASC, CAMH), Table 3 presents the profile prevalence estimates of the six profiles. Additionally, the CASC and CAMH estimates are combined into estimates for adolescents in care.

#### Community versus in care

The ‘no difficulties’ profile was estimated to be 11 times more prevalent among community setting adolescents than among adolescents in care (55 and 5%, respectively). In contrast, the five profiles indicating the presence of at least a single type of difficulties were jointly estimated to be over two times more prevalent among adolescents in care than among community setting adolescents (95 and 45%, respectively). For these five profiles, the main differences between community setting adolescents and adolescents in care were found for the profiles with mean scores in the ‘abnormal’ range: ‘emotional difficulties’ (community: 9%, in care: 20%), ‘emotional and social difficulties’ (community: 4%, in care: 21%), and ‘overall difficulties’ (community: 1%, in care: 20%).

#### CASC versus CAMH

Differences in prevalence estimates between CASC and CAMH are found for four of the five profiles indicating the presence of difficulties: The ‘borderline hyperactivity difficulties’ (CASC: 34%; CAMH 16%) and ‘overall difficulties’ (CASC: 31%, CAMH: 20%) profiles were estimated to be more prevalent among adolescents receiving CASC and the ‘emotional difficulties’ (CASC: 8%; CAMH: 20%) and ‘emotional and social difficulties’ (CASC: 7%; CAMH 22%) profiles more prevalent among adolescents receiving CAMH.

#### Gender differences

Among adolescents in care, a few gender differences ≥ 20% were found. Males showed a higher estimated prevalence for the ‘borderline conduct and social difficulties’ (males: 57%; females: 20%) profile within the CASC setting. Females showed higher prevalence estimates for ‘borderline hyperactivity difficulties’ (males: 4%; females: 27%) within the CASC setting and ‘emotional difficulties’ (males: 8%; females: 32%) and ‘emotional and social difficulties’ (males: 10%; females: 32%) within the CAMH setting.

### Obtaining a preliminary indication of disorders

For adolescents within the CAMH setting, Table 4 presents the prevalence estimates of the six common SDQ profiles per DSM-IV diagnosis, including combinations of diagnoses. Per disorder (combination), the percentages for content-wise matching SDQ profiles are printed in bold. In total, for 88% of the diagnosed adolescents the DSM-IV diagnoses matched the reported types of difficulties.

#### Anxiety/Mood disorder, and additional diagnoses

As shown in Table 4, 86% of adolescents diagnosed with only Anxiety/Mood disorder was estimated to have one of the content-wise matching SDQ profiles (‘emotional difficulties’: 39%; ‘emotional and social difficulties’: 38%; ‘overall difficulties’: 9%). Compared to adolescents diagnosed with only Anxiety/Mood disorder, adolescents with an additional ADHD disorder showed higher prevalence estimates for ‘borderline hyperactivity difficulties’ (5 versus 20%, respectively) and ‘overall difficulties’ (9 versus 26%, respectively), and a lower estimate for ‘emotional difficulties’ (39 versus 20%, respectively). Adolescents additionally diagnosed with ASD showed a higher estimate for ‘emotional and social difficulties’ (38 versus 66%, respectively) and a lower estimate for ‘emotional difficulties’ (39 versus 17%, respectively) than adolescents diagnosed with only Anxiety/Mood disorders did.

#### CD/ODD, and additional diagnoses

Among adolescents diagnosed with only CD/ODD, 68% was estimated to have one of the content-wise matching profiles (‘borderline conduct and social difficulties’: 35%; ‘overall difficulties’: 33%). Compared to adolescents diagnosed with only CD/ODD, adolescents additionally diagnosed with ADHD showed higher prevalence estimates for ‘overall difficulties’ (33 versus 58%, respectively) and ‘borderline hyperactivity difficulties’ (22 versus 36%, respectively), and a lower estimate for ‘borderline conduct and social difficulties’ (35 versus 6%, respectively).

#### ADHD, and additional diagnoses

Among adolescents diagnosed with only ADHD, 86% was estimated to have a content-wise matching SDQ profile (‘borderline hyperactivity difficulties’: overall: 57%, males: 65%, females: 41%; ‘overall difficulties’: 29%). Compared to adolescents diagnosed with only ADHD, adolescents with an additional Anxiety/Mood diagnosis showed higher prevalence estimates for ‘emotional difficulties’ (4 versus 20%, respectively) and ‘emotional and social difficulties’ (6 versus 32%, respectively), and a lower estimate for ‘borderline hyperactivity difficulties’ (57 versus 20%, respectively). Adolescents additionally diagnosed with CD/ODD showed a higher estimate for ‘overall difficulties’ (29 versus 58%, respectively) and a lower estimate for ‘borderline hyperactivity difficulties’ (57 versus 36%, respectively) than adolescents diagnosed with only ADHD did. Adolescents with an additional ASD diagnosis showed higher estimates for ‘borderline conduct and social difficulties’ (2 versus 21%, respectively) and ‘overall difficulties’ (29 versus 50%, respectively), and a lower estimate for ‘borderline hyperactivity difficulties’ (57 versus 10%, respectively).

#### ASD, and additional diagnoses

For adolescents diagnosed with only ASD, 91% was estimated to have a content-wise matching SDQ profile (‘borderline conduct and social difficulties’: overall: 42%, among males: 50%, among females: 26%; ‘emotional and social difficulties’: 28%; ‘overall difficulties’: 21%). Compared to adolescents diagnosed with only ASD, adolescents with an additional Anxiety/Mood disorder diagnosis showed a higher prevalence estimate for ‘emotional and social difficulties’ (28 versus 66%, respectively) and a lower estimate for ‘borderline conduct and social difficulties’ (42 versus 7%, respectively). Adolescents additionally diagnosed with ADHD showed a higher estimate for ‘overall difficulties’ (21 versus 50%, respectively) and a lower estimate for ‘borderline conduct and social difficulties’ (42 versus 21%, respectively) than adolescents diagnosed with only ASD did.

#### Other or no diagnoses

For adolescents receiving CAMH that are diagnosed with DSM-IV disorders, other than Anxiety/Mood, CD/ODD, ADHD and ASD, the highest profile prevalence estimate was found for the ‘emotional difficulties’ profile (overall: 36%; among males: 16%; among females: 45%). The probabilities for the remaining profiles were lower and fairly equal to each other (i.e. between 10 and 16%).

### Multi informants versus single informant

Regarding informants, Fig. 1 and Table S4 (available online) show that the adolescents themselves did not indicate the presence of difficulties for the ‘borderline conduct and social difficulties’ and the ‘emotional difficulties’ SDQ profiles, whereas the parents did for one or two difficulties subscales per profile. Based on only adolescent self-report, these two profiles would have merged with the ‘no difficulties’ profile. This would have resulted in ‘no difficulties’ being much more prevalent: 81% among adolescents not in care (55% with the profile approach based on both informants, see Table 3) and 41% among adolescents in care (5% with the profile approach based on both informants, see Table 3).

### SDQ profiles versus the total difficulties scale

Table 5 shows that 58% (self-reported SDQ version; 2386 of 4130) and 37% (parent-reported SDQ version; 1500 of 4020) of adolescents in care showed a total difficulties scale score within the ‘normal’ range. Thus, compared to the profile approach, the total difficulties scale would have indicated the presence of problems among substantially fewer adolescents in care: 42% with the self-reported SDQ version and 63% with the parent-reported SDQ version (95% based on the profile approach, see Table 3). The most prevalent profiles among adolescents in care with ‘normal’ total difficulties scale scores are the ‘borderline hyperactivity difficulties’, ‘borderline conduct and social difficulties’ and the ‘emotional difficulties’ profiles.

**Table 5:** SDQ profile prevalence estimates in percentages per self-reported and parent-reported total difficulties scale score classification (‘normal’, ‘borderline’/’abnormal’) in the community, CASC and CAMH settings

	Total difficulties scale score classification	*N *(% within setting)	SDQ profile					
			No difficulties	Borderline hyperactivity difficulties	Borderline conduct and social difficulties	Emotional difficulties	Emotional and social difficulties	Overall difficulties
			% All (M/F)^a^	% All (M/F)^a^	% All (M/F)^a^	% All (M/F)^a^	% All (M/F)^a^	% All (M/F)^a^
Setting	*Self-reported SDQ version*							
Community	‘Normal’	1106 (90)	64	10	19	7	0	0
	‘Borderline’/‘abnormal’	118 (10)	10	13	3	5	52 (35/69)	16
In care (total)	‘Normal’	2386 (58)	10	24	32 (42/20)	22 (7/40)	5	6
	‘Borderline’/‘abnormal’	1744 (42)	0	3	1	5	52 (30/64)	39 (62/26)
CASC	‘Normal’	53 (57)	90	1	8	1	0	0
	‘Borderline’/‘abnormal’	40 (43)	33	16	16	1	37 (15/51)	27
CAMH	‘Normal’	2,333 (58)	12	25	29 (38/17)	22 (7/41)	5	7
	‘Borderline’/‘abnormal’	1,704 (42)	1	3	1	5	53 (32/65)	38 (60/25)
	*Parent-reported SDQ version*							
Community	‘Normal’	731 (87)	76	16	0	8	0	3
	‘Borderline’/‘abnormal’	110 (13)	0	0	47 (62/22)	0	42 (24/70)	11
In care (total)	‘Normal’	1500 (37)	19	25 (39/12)	7	44 (18/64)	4	3
	‘Borderline’/‘abnormal’	2520 (63)	0	7	17	4	35 (17/53)	37 (47/27)
CASC	‘Normal’	34 (32)	95	5	0	1	0	0
	‘Borderline’/‘abnormal’	71 (68)	2	0	46 (54/30)	0	17 (6/30)	35
CAMH	‘Normal’	1466 (37)	21	25 (40/12)	3	45 (19/65)	4	2
	‘Borderline’/‘abnormal’	2449 (63)	1	6	17	4	36 (18/55)	36 (46/26)

*SDQ *Strengths and Difficulties Questionnaire, *CASC *Child and Adolescent Social Care, *CAMH* Child and Adolescent Mental Healthcare

^a^Profile prevalence estimates in percentages for males and females are reported for gender differences > 20%

### SDQ profiles versus the SDQ scoring algorithm

To further assess the usefulness of the SDQ score profile approach (profile approach in what follows), the SDQ scoring algorithm (algorithm for short in what follows) that is available on the SDQ website was applied to the scores of adolescents within the CAMH sample. The two approaches were compared in terms of how well their results matched the disorders diagnosed among the adolescents. Note that the approaches could only be compared to a very limited extent, as the algorithm does not include the social difficulties scale and the prosocial behaviour scale, meaning that the algorithm cannot be used to predict the presence of ASD as a single disorder or as part of a combination of disorders. The results of the algorithm and the profile approach are summarized in Table S7 (available online). Among adolescents diagnosed with *a single disorder*, the algorithm and the profile approach produced fairly similar results regarding Anxiety/Mood disorder, CD/ODD and ADHD. Regarding adolescents diagnosed with *multiple disorders*, the results of the algorithm and the profile approach are much harder to compare as the algorithm results in predictions for specific disorders and not for comorbidity of disorders, whereas half of the profiles identified in this study indicate the presence of multiple types of difficulties. The online materials contain a more detailed description of the results.

## Discussion

Up to now knowledge was lacking on how the rich information on multiple problem domains contained in the SDQ rated by multiple informants can be used for screening. We addressed this topic by assessing the validity of using adolescents’ SDQ profiles that combined all self- and parent-rated SDQ subscale information for screening, rather than only separate subscales or total difficulties scores reported by a single informant. Our findings show that the SDQ profile approach is useful for screening, as the profiles were found to be associated with care use, CASC as well as CAMH, and type of diagnosed DSM-IV disorder. Moreover, the SDQ profile approach was found to be more useful for screening than (a) a single-informant profile approach, especially if that single informant is the adolescent, and (b) using only the total difficulties scale. The validity of using SDQ profiles partly differed for male and female adolescents. Additionally, the profile SDQ profile approach was found to be at least equally useful for indicating the potential presence of disorders as the SDQ scoring algorithm was, especially among adolescents diagnosed with a single disorder or combinations of disorders that include ASD.

Finding that the SDQ profile approach is more useful for screening than a single-informant profile approach, especially if that single informant is the adolescent, adds in various ways to previous research. Previous research focusing on distinguishing between adolescents from general and mental healthcare populations showed ratings from both informants to be independently useful for this purpose [9–11], whereas our findings show that the value of adolescent ratings depends on the type and/or severity of problems present. Moreover, our findings add evidence regarding the unclear value of adolescent self- and parent-rated SDQ information for obtaining a preliminary indication of the presence of Anxiety/Mood disorder [14–16] by finding the parent to be an important informant for indicating the presence of Anxiety/Mood disorder. As self-report is commonly regarded as more accurate for internalizing problems [4, 25], it is a somewhat surprising finding. A potential explanation may lie in the fact that the samples from the CASC and CAMH settings consist of referred adolescents. Our finding could merely reflect the known phenomenon that during adolescence parent-reported need for care exceeds adolescent-reported need for care [26].

The finding that the SDQ profile approach is more useful for screening than only the total difficulties scale, contrasts with previous findings on the value of the total difficulties scale for distinguishing between adolescents from general and mental healthcare populations. This previous research showed that the adolescent self- and parent-rated total difficulties scales were separately useful for that purpose [9–11], whereas we found the SDQ total difficulties scale to insufficiently reflect specific psychiatric problems. That is, a substantial number of adolescents whose SDQ subscale scores indicated the presence of borderline problem severity or emotional difficulties would have been overlooked based on their total difficulties scale scores. This finding is not surprising as it makes sense that problems in one or a few domains does not amount to an increased score on the total problems scale.

In addition, for all types of single DSM-IV diagnoses we found small, yet non-zero prevalence estimates for profiles with types of reported difficulties that did not match the DSM-IV diagnosis involved. We interpret this as an illustration of a well-known phenomenon in informant reports [27, 28]: the intentional or accidental underreporting, over-reporting, or misreporting of problems. Although the DSM-IV diagnoses undoubtedly also have errors and partial content overlap and the findings of this study generally support the use of the SDQ for screening, these additional findings emphasize the widely acknowledged limit of using questionnaires as the sole instrument for diagnosing [29].

The comparison between the profile approach and the scoring algorithm approach indicated that the algorithm and the profile approach are equally useful for indicating the potential presence of single disorders for Anxiety/Mood disorder CD/ODD, and ADHD. As the social and prosocial scales are not used in the scoring algorithm, the profile approach is more useful than the algorithm approach for indicating the potential presence of ASD as single disorder and as part of a combination of disorders. For other combinations of disorders, the two approaches could not be compared meaningfully, because the algorithm provides prediction per type of disorder and not for combinations of disorders.

### Implications

Our findings support the combined use of self- and parent-rated SDQ subscales for (a) distinguishing between adolescents in care and adolescents not in care and (b) providing a preliminary indication of the disorders present. We advise against the use of only the SDQ total difficulties scale for screening, as our findings imply that this will result in a substantial number of adolescents with reported problems on the SDQ subscales being overlooked. Our findings further suggest that for screening purposes the parent is more useful as single informant than the adolescent is.

Our exploration regarding gender differences in the validity of using adolescents’ SDQ profiles for screening implies that screening accuracy can be improved by applying gender-specific cutoffs for interpreting SDQ scale scores, as internalizing DSM-IV diagnoses were insufficiently reflected in SDQ scores for males, and externalizing diagnoses were insufficiently reflected in SDQ scores for females. It is commonly known that certain behaviors are displayed more frequently or are more outspoken among males than females, and vice versa [17, 30]. As this brings about a risk of under-diagnosis of females and males, respectively, we presume that it is of interest to identify adolescents with relatively extreme behavior compared to other adolescents of the same gender. To facilitate such comparisons, further research is needed to obtain gender-specific cutoff values based on representative samples of adolescents. This could be achieved through regression-based norming [31]. The availability of gender-specific cutoffs would be consistent with current practice for other questionnaires measuring behavior, such as the Child Behavior Checklist [32] and its self-report version the Youth Self Report [33].

In addition, our findings regarding the SDQ score profile approach match with the proposal to combine the emotional difficulties scale and the social difficulties scale into an internalizing difficulties scale, to indicate the potential presence of emotional disorders and ASD [34]. That is, we identified an ‘emotional and social difficulties’ profile that was highly prevalent among adolescents diagnosed with these disorders.

Further, our findings imply that clinicians should be provided with instructions, preferably included in an (online) automated scoring tool, on how to obtain an individual adolescent’s probability for displaying each of the common SDQ score profiles, and subsequently obtaining estimates on which disorder(s) are possibly present. We were able to provide information for estimating an adolescent’s probabilities for each of the six profiles identified in this study. These probabilities yield some uncertainty, because the samples used in this study are not random samples from their respective populations. For that same reason, and because the prevalence of disorders in the population of adolescents is unknown to us, we could not provide information for obtaining probability estimates on which disorder(s) are possibly present. These matters require further research using random samples and/or the availability of information on the prevalence of disorders.

Besides, it is important to keep in mind that the SDQ was not developed for diagnostic purposes or for evaluating the need for care. Adolescents with a ‘no difficulties’ SDQ score profile may be in need of care (e.g., because of a risk-bearing parenting setting), and adolescents profiles that indicate the presence of difficulties in the ‘borderline’ or even ‘abnormal’ range may not be in need of care (e.g. watchful waiting in the presence of a supporting family environment may sometimes be the better option). The SDQ can be used as a preliminary indication of potential problems, with a diagnostic assessment by clinicians thereafter.

### Strengths and limitations

The main strengths of our study are that our findings are based on samples of substantial sizes and that our clinical sample consisted of adolescents with a large variety of mental health problems, yielding a relatively low risk of uncertainty due to sampling fluctuation in our estimations and a relatively high probability that our sample covers the types and severity of problems in the Dutch clinical population. The main limitations of our study were that our samples were not random samples from their respective populations. Earlier we mentioned that the community setting sample and potentially the sample from the CAMH setting are not fully representative of their respective Dutch adolescent populations. Consequently, we do not know how well the profiles found in this study represent the profiles prevalent in the populations these samples we drawn from. Besides, we used the British cutoff scores to label the profiles, while it is unknown whether they hold for the Dutch adolescent population [16]. Norms for Dutch adolescents are available [35, 36]. Unfortunately, these norms are based on relatively small samples that are indicated as possibly not representative by the researchers who established the norms. As a consequence, we are insufficiently confident of their usefulness for the purpose of this study. Moreover, although we acknowledge the value of the impact scale for clinical practice, we did not include it in our current analyses. Whether or not the profile approach can possibly benefit from a meaningful inclusion of this scale requires further research.

### Conclusion

This study provides four main insights for the use of the SDQ in practice: (1) the SDQ profiles that combine adolescent self- and parent-rated subscale scores are useful for screening, (2) more so than SDQ scale scores reported by a single informant, and (3) more so than using the total difficulties scale. This profile approach can help practitioners put information on multiple problem domains rated by multiple informants to better use for the benefit of adolescents. The usefulness of SDQ profiles for screening can be enhanced by (4) using gender-specific cutoffs, as was indicated by exploratory analyses.

## Electronic supplementary material

Below is the link to the electronic supplementary material.

Supplementary file1 (PDF 241 kb)

## Data Availability

Due to third party restrictions, we cannot share our data publicly. Upon reasonable request, the corresponding author can share part of the data and put the requestor in contact with the third parties.
