# Supplementary material for: The combined self- and parent-rated SDQ score profile predicts care use and psychiatric diagnoses
Source: Eur Child Adolesc Psychiatry. 2020 Oct 30;30(12):1983–94. doi: 10.1007/s00787-020-01667-5 (PMC8563533; doi:10.1007/s00787-020-01667-5)
Supplement: Supplementary file 1 — Supplementary file1 (PDF 241 kb) [file 787_2020_1667_MOESM1_ESM.pdf]

Supplementary tables

Material supplementary to:

The combined self- and parent-rated SDQ score profile predicts care use and psychiatric diagnoses. European Child & Adolescent Psychiatry

Authors: J. Vugteveen[1], A. de Bildt, C.A. Hartman, S.A. Reijneveld, M.E. Timmerman

[1] Heymans Institute for Psychological Research, University of Groningen, the Netherlands, Email: j.vugteveen@rug.nl

Table S1 Mean SDQ scale scores and standard deviations per informant and setting

	Community		CASC		CAMH	
SDQ scale	Adolescent ($N = 1217$)	Parent ($N = 839$)	Adolescent ($N = 93$)	Parent ($N = 105$)	Adolescent ($N = 4037$)	Parent ($N = 3915$)
Total	8.6 (5.0)*	7.0 (5.6)*	14.4 (5.9)	16.7 (6.8)	14.5 (5.9)*	15.9 (6.5)*
Emotional	2.3 (2.1)*	1.9 (2.1)*	3.8 (2.7)	3.9 (2.4)	4.4 (2.8)*	5.0 (2.8)*
Conduct	1.4 (1.3)*	1.0 (1.4)*	3.0 (1.9)*	4.5 (2.5)*	2.6 (1.8)*	2.8 (2.4)*
Hyperactivity/ inattention	3.6 (2.4)*	2.7 (2.5)*	5.2 (2.7)	5.4 (2.7)	5.3 (2.6)	5.2 (2.8)
Social	1.4 (1.6)	1.5 (1.8)	2.4 (1.8)	2.9 (2.2)	2.3 (1.9)*	2.9 (2.3)*
Prosocial	8.0 (1.7)*	8.3 (1.8)*	7.7 (1.8)	6.5 (2.3)	7.9 (1.8)[a]*	7.4 (2.2)*
Impact	0.9 (1.9)	0.9 (2.1)	3.4 (3.1)*	5.5 (3.3)*	2.4 (2.4)*	3.8 (2.6)*

SDQ = Strengths and Difficulties Questionnaire, CASC = child and adolescent social care, CAMH = child and adolescent mental health

[a] $N = 4036$

* Informant difference significant at $\alpha = .01$ (Bonferroni correction applied per setting)

Table S2 Per setting, SDQ profile prevalence estimates in percentages

		SDQ profile					
Setting	Gender	No difficulties	Borderline hyperactivity difficulties	Borderline conduct and social difficulties	Emotional difficulties	Emotional and social difficulties	Overall difficulties
Community	% All (*M/F)*	55 (*52/58*)	15 (18/*12*)	17 (*24/11*)	9 (*1/16*)	4 (*3/4*)	1 (*2/0*)
In Care (total)	% All (*M/F)*	5 (*6/5*)	18 (*26/1*)	16 (*24/9*)	20 (*8/31*)	21 (*11/31*)	20 (*25/14*)
CASC	% All (*M/F)*	2 (*0/3*)	18 (*4/27*)	34 (*57/20*)	8 (*0/14*)	7 (*0/11*)	31 (*39/26*)
CAMH	% All (*M/F)*	5 (*6/4*)	18 (*26/10*)	16 (*24/8*)	20 (*8/32*)	22 (*11/32*)	20 (*25/14*)

SDQ = Strengths and Difficulties Questionnaire; M = male adolescents, F = female adolescents.

Table S3 Per DSM-IV diagnosis (or combination of diagnoses), SDQ profile prevalence estimates in percentages among child and adolescent mental healthcare (CAMH) adolescents

		SDQ profile					
DSM-IV diagnosis	Gender	No difficulties	Borderline hyperactivity difficulties	Borderline conduct and social difficulties	Emotional difficulties	Emotional and social difficulties	Overall difficulties
Anxiety/Mood	% All (*M/F*)	3 (*6/2*)	5 (*9/3*)	6 (*18/2*)	39 (*31/42*)	38 (*25/42*)	9 (*11/9*)
CD/ODD	% All (*M/F*)	4 (*6/0*)	22 (*24/19*)	35 (*39/28*)	2 (*0/6*)	3 (*0/9*)	33 (*31/38*)
ADHD	% All (*M/F*)	3 (*4/1*)	57 (*65/41*)	2 (*2/0*)	4 (*0/12*)	6 (*1/16*)	29 (*28/30*)
ASD	% All (*M/F*)	2 (*2/1*)	1 (*1/0*)	42 (*50/26*)	7 (*2/15*)	28 (*22/39*)	21 (*23/19*)
Anxiety/Mood & ADHD	% All (*M/F*)	1 (*0/1*)	20 (*40/8*)	0 (*0/0*)	20 (*9/28*)	32 (*23/38*)	26 (*29/25*)
Anxiety/Mood & ASD	% All (*M/F*)	0 (*0/0*)	0 (*0/0*)	7 (*16/2*)	17 (*19/14*)	66 (*50/80*)	10 (*15/6*)
CD/ODD & ADHD	% All (*M/F*)	0 (*0/0*)	36 (*33/48*)	6 (*7/1*)	0 (*0/0*)	0 (*0/0*)	58 (*60/52*)
ADHD & ASD	% All (*M/F*)	2 (*2/0*)	10 (*10/9*)	21(*26/1*)	0 (*0/0*)	17 (*18/15*)	50 (*44/75*)
Other[a]	% All (*M/F*)	10 (*13/9*)	15 (*25/10*)	13 (*22/9*)	36 (*16/45*)	16 (*5/21*)	10 (*20/6*)
Diagnosis unavailable[b]	% All (*M/F*)	11 (*13/10*)	23 (*28/17*)	23 (*30/16*)	13 (*5/22*)	12 (*4/20*)	18 (*21/15*)

SDQ = Strengths and Difficulties Questionnaire, M = male adolescents, F = female adolescents, CD/ODD = conduct / oppositional defiant disorder, ADHD = Attention deficit hyperactivity disorder, ASD = Autism spectrum disoder

[a] Adolescents diagnosed with DSM-IV disorders other than ADHD, CD/ODD, Anxiety/Mood disorder, ASD

[b] Adolescents for whom no diagnosis was established or the information was not registered adequately

Table S4 Per SDQ profile, mean scale scores and standard deviations for both SDQ versions

		SDQ scale									
		Emotional		Conduct		Hyperactivity/inattention		Social		Prosocial	
SDQ profile	Estimated proportion	A	P	A	P	A	P	A	P	A	P
		M (SD)	*M (SD)*	*M (SD)*	*M (SD)*	*M (SD)*	*M (SD)*	*M (SD)*	*M (SD)*	*M (SD)*	*M (SD)*
No diff.[a]	.16	1.5 (1.5)	1.1† (1.3)	0.8 (0.9)	0.4† (0.6)	2.7 (2.1)	1.8† (1.8)	0.8 (1.1)	0.8 (1.1)	8.5 (1.5)	9.0† (1.2)
Borderline hyperactivity diff.[b]	.17	2.6 (2.0)	3.0† (2.2)	2.6 (1.6)	2.7 (1.6)	6.2* (2.1)	6.2* (2.1)	1.0 (1.1)	1.0 (1.1)	8.0 (1.6)	7.8 (1.7)
Borderline conduct and social diff.[b]	.17	2.2 (1.8)	3.7† (2.3)	2.0 (1.6)	3.2*† (2.1)	3.4 (2.1)	4.6† (2.2)	2.1 (1.6)	3.7*† (2.0)	7.4 (1.8)	6.0*† (2.1)
Emotional diff.[c]	.17	5.6 (2.1)	5.5** (2.1)	1.2 (1.1)	0.8† (1.0)	4.0 (2.2)	2.9† (2.1)	2.2 (1.7)	2.0 (1.6)	8.8 (1.3)	9.0 (1.2)
Emotional and social diff.[c]	.17	6.9* (2.0)	7.2** (2.0)	2.7 (1.5)	2.2† (1.4)	5.8 (2.2)	5.0† (2.3)	3.8 (1.9)	4.4**† (2.0)	7.7 (1.9)	7.4 (2.0)
Overall diff.[c]	.16	4.4 (2.5)	5.6**† (2.5)	4.6* (1.7)	5.7**† (1.9)	7.2** (1.9)	7.7**† (1.8)	2.8 (2.0)	4.1**† (2.4)	6.8 (2.0)	5.8*† (2.2)

SDQ = Strengths and Difficulties Questionnaire, A = adolescent self-reported SDQ version, P = parent-reported SDQ version, *M (SD)* = mean (standard deviation), diff. = difficulties

[a] SDQ profile containing scores in the ‘normal’ range all subscales

[b] SDQ profile containing scores in the ‘borderline’ range all subscales

[c] SDQ profile containing scores in the ‘abnormal’ range on one or more subscales

* (Sub)scale score in the ‘borderline’ range according to the informant-dependent British cutoffs

** (Sub)scale score in the ‘abnormal’ range according to the informant-dependent British cutoffs

† Informant differences significant at the α = .0016 level (Bonferroni correction applied per cluster)

Table S4 (continued) Per SDQ profile, mean scale scores and standard deviations for both SDQ versions

	SDQ scale			
	Impact		Total	
SDQ profile	A	P	A	P
	M (SD)	*M (SD)*	*M (SD)*	*M (SD)*
No diff.[a]	0.4 (1.0)	0.5 (1.2)	5.8 (7.1)	4.1† (6.0)
Borderline hyperactivity diff.[b]	1.6 (1.9) *	2.6 (2.1) † **	12.4 (8.4)	12.9 (8.7)
Borderline conduct and social diff.[b]	1.1 (1.7) *	3.5 (2.6) † **	9.7 (8.9)	15.2*† (10.7)
Emotional diff.[c]	2.4 (2.4) **	2.9 (2.6) † **	13.0 (8.4)	11.2† (8.0)
Emotional and social diff.[c]	4.0 (2.6) **	5.0 (2.6) † **	19.2 (9.5) *	18.8 (9.7) **
Overall diff.[c]	2.8 (2.4) **	5.2 (2.5) † **	19.0 (10.1)*	23.1† (10.8) **

SDQ = Strengths and Difficulties Questionnaire, A = adolescent self-reported SDQ version, P = parent-reported SDQ version, diff. = difficulties

[a] SDQ profile containing scores in the 'normal' range all subscales

[b] SDQ profile containing scores in the 'borderline' range all subscales

[c] SDQ profile containing scores in the 'abnormal' range on one or more subscales

* (Sub)scale score in the 'borderline' range according to the informant-dependent British cutoffs

** (Sub)scale score in the 'abnormal' range according to the informant-dependent British cutoffs

† Informant differences significant at the α = .0016 level (Bonferroni correction applied per cluster)

Table S5 Per SDQ profile, mean subscale scores for both SDQ versions (based on community sample data only)

		SDQ subscale									
		Emotional		Conduct		Hyperactivity		Social		Prosocial	
SDQ profile	Estimated proportion	A	P	A	P	A	P	A	P	A	P
Super healthy[a]	.52	1.7	0.9	0.7	0.3	2.6	1.4	0.8	0.8	8.5	9.0
Healthy[a]	.48	2.9	2.9	2.1	1.7	4.7	4.1	2.0	2.3	7.4	7.5

SDQ = Strengths and Difficulties Questionnaire, A = adolescent self-reported SDQ version, P = parent-reported SDQ version

[a] SDQ profile containing scores in the 'normal' range all subscales

Table S6 Per SDQ profile, mean subscale scores for both SDQ versions (based on CAMH sample data only)

		SDQ subscale									
		Emotional		Conduct		Hyperactivity		Social		Prosocial	
SDQ profile	Estimated proportion	A	P	A	P	A	P	A	P	A	P
No difficulties[a]	.15	2.7	2.6	1.2	0.6	3.6	3.1	1.4	1.3	8.8	9.1
Borderline hyperactivity and conduct difficulties[b]	.17	2.6	3.4	3.2	3.5*	6.7*	7.0*	1.0	1.2	7.8	7.3
Borderline emotional, conduct and social difficulties[b]	.17	2.2	4.0*	2.1	3.4*	3.5	4.8	2.0	3.7*	7.6	5.9*
Emotional difficulties[c]	.18	6.4*	6.4**	1.5	1.1	4.4	3.3	2.2	1.9	8.7	8.8
Emotional and social difficulties[c]	.18	6.9*	7.2**	2.6	2.2	5.7	4.9	4.0*	4.7**	7.7	7.3
Overall difficulties[c]	.16	4.9	6.1**	4.7*	5.7**	7.4**	7.7**	3.1	4.5**	6.8	5.8*

SDQ = Strengths and Difficulties Questionnaire, A = adolescent self-reported SDQ version, P = parent-reported SDQ version

[a] SDQ profile containing scores in the ‘normal’ range all subscales

[b] SDQ profile containing scores in the ‘borderline’ range all subscales

[c] SDQ profile containing scores in the ‘abnormal’ range on one or more subscales

* Subscale score in the ‘borderline’ range according to the informant-dependent British cutoffs

** Subscale score in the ‘abnormal’ range according to the informant-dependent British cutoffs

The SDQ scoring algorithm compared to the SDQ score profile approach

To compare the SDQ scoring algorithm to the SDQ score profile approach, both have been applied to diagnosed adolescents from the CAMH sample. The adolescents have been diagnosed with Anxiety/Mood disorder, CD/ODD, ADHD, or ASD (or combinations thereof). The SDQ scoring algorithm results in 'unlikely', 'possible' or 'probable' predictions for emotional disorders, conduct disorders, and hyperactivity disorders. Additionally, the algorithm provides such predictions for the presence of any of these disorders. For this comparison of approaches, we consider both 'possible' and 'probable' predictions to indicate the potential presence of a disorder. The profile approach results in the estimated prevalence of six profiles per diagnosis group. The content of these profiles can either match the diagnosed disorder or not (e.g., the 'emotional difficulties' profile matches Anxiety/Mood disorder, but not CD/ODD).

Results

The results of the SDQ scoring algorithm (algorithm for short in what follows) and the SDQ profile approach (profile for short in what follows) are summarized in Table S7. From this table, the following can be derived.

Anxiety/Mood disorder, and additional diagnoses. The algorithm showed the potential presence of an emotional disorder among 78% of adolescents diagnosed with Anxiety/Mood disorder, whereas the profile approach resulted in the estimated presence of emotional difficulties among 80% of these adolescents. Among adolescents diagnosed with Anxiety/Mood disorder and ADHD, the algorithm predicted the potential presence of an emotional disorder among 78%, and the potential presence of a hyperactivity disorder among 66%. The profiles indicated the presence of emotional difficulties and/or hyperactivity difficulties among 91%. Among adolescents diagnosed with Anxiety/Mood disorder and ASD, the algorithm predicted the potential presence of an emotional disorder among 89%. The algorithm does not result in a prediction for ASD. The profiles indicated the presence of emotional and/or social difficulties among 86%.

CD/ODD, and additional diagnoses. The algorithm showed the potential presence of a conduct disorder among 65% of adolescents diagnosed with just CD/ODD, whereas the profile

approach resulted in the estimated presence of conduct difficulties among 61% of these adolescents. Among adolescents diagnosed with CD/ODD and ADHD, the algorithm predicted the potential presence of a conduct disorder among 79%, and the potential presence of a hyperactivity disorder among 72%. The profiles indicated the presence of conduct and/or hyperactivity difficulties among 94%.

ADHD, and additional diagnoses. The algorithm showed the potential presence of a hyperactivity disorder among 73% of adolescents diagnosed with just ADHD, whereas the profile approach resulted in the estimated presence of hyperactivity difficulties among 70% of these adolescents. Among adolescents diagnosed with ADHD and Anxiety/mood disorder, the algorithm predicted the potential presence of a hyperactivity disorder among 66%, and the potential presence of an emotional disorder among 78%. The profiles indicated the presence of hyperactivity and/or emotional difficulties among 91%. Among adolescents diagnosed with ADHD and CD/ODD, the algorithm predicted the potential presence of a hyperactivity disorder among 72%, and the potential presence of a conduct disorder among 79%. The profiles indicated the presence of conduct difficulties and/or hyperactivity difficulties among 94%. Finally, among adolescents diagnosed with ADHD disorder and ASD, the algorithm predicted the potential presence of a hyperactivity disorder among 77%. The algorithm does not result in a prediction for ASD. The profiles indicated the presence of hyperactivity and/or social difficulties among 94%.

Conclusion

Among adolescents diagnosed with *a single disorder*, the results described above show that algorithm approach and the profile approach produced fairly similar results regarding Anxiety/Mood disorder, CD/ODD and ADHD. For ASD, the results of the two approaches cannot be compared as the algorithm does not provide an indication of the potential presence of a social disorder.

Regarding adolescents diagnosed with *multiple disorders*, the results of the algorithm and the profile approach are much harder to compare as the algorithm results in predictions for specific disorders and not for comorbidity of disorders, whereas half of the profiles identified in this study indicate the presence of multiple types of difficulties. Besides, half of the disorder combinations

considered in this study involve social disorders (i.e., ASD), for which the algorithm does not provide predictions.

Assuming that the diagnoses received by the adolescents in the CAMH sample are valid, these findings indicate that the algorithm and the profile approach are about equally useful for predicting single disorders Anxiety/Mood disorder, CD/ODD and ADHD. Regarding the prediction of combinations of disorders, no sensible comparison could be made between the two approaches. Needless to say, the profile approach is more useful than the algorithm approach for indicating the potential presence of ASD as single disorder and as part of a combination of disorders.

Table S7 Per disorder (combination) diagnosed among CAMH adolescents, a summary of the SDQ scoring algorithm classification and the SDQ score profile results in number of adolescents (and percentages for the totals)

	SDQ scoring algorithm		SDQ score profile							
DSM-IV diagnosis	Predicted type of disorder	Prediction	No difficulties	Borderline hyperactivity difficulties	Borderline conduct and social difficulties	Emotional difficulties	Emotional and social difficulties	Overall difficulties	*N*	%
Anxiety/mood	Emotional	Possible/probable	3	40	48	317	384	95	887	78
		Unlikely	47	44	49	84	14	14	252	22
		Total (%[a])	50 (4)	48 (4)	97 (9)	401 (35)	398 (35)	109 (10)	1.139	
CD/ODD	Conduct	Possible/probable	0	23	39	0	7	56	125	65
		Unlikely	11	20	18	8	7	3	67	35
		Total (%)	11 (6)	43 (22)	57 (30)	8 (4)	14 (7)	59 (31)	192	
ADHD	Hyperactivity	Possible/probable	8	184	26	12	33	130	393	73
		Unlikely	22	52	19	22	16	12	143	27
		Total (%)	30 (6)	236 (44)	45 (8)	34 (6)	49 (9)	142 (26)	536	
ASD	n/a	n/a	n/a	n/a	n/a	n/a	n/a	n/a	n/a	n/a
		Total (%)	20 (4)	39 (8)	148 (30)	47 (10)	129 (27)	103 (21)	486	
Anxiety/Mood & ADHD	Emotional	Possible/probable	1	7	2	18	30	22	80	78
		Unlikely	3	12	2	2	1	3	23	22
	Hyperactivity	Possible/probable	1	17	3	4	19	24	68	66
		Unlikely	3	2	1	16	12	1	35	34
		Total (%)	4 (4)	19 (18)	4 (4)	20 (19)	31 (30)	25 (24)	103	
Anxiety/Mood & ASD	Emotional	Possible/probable	0	2	5	19	59	14	99	89
		Unlikely	0	3	6	3	0	0	12	11

		Total (%)	0 (0)	5 (5)	11 (10)	22 (20)	59 (53)	14 (13)	111	
CD/ODD & ADHD	Conduct	Possible/probable	0	28	9	0	1	70	108	79
		Unlikely	2	13	6	3	3	2	29	21
	Hyperactivity	Possible/probable	0	21	5	2	3	67	98	72
		Unlikely	2	20	10	1	1	5	39	28
		Total (%)	2 (1)	41 (30)	15 (11)	3 (2)	4 (3)	72 (53)	137	
ADHD & ASD	Hyperactivity	Possible/probable	0	13	10	0	16	45	84	77
		Unlikely	2	2	9	4	4	4	25	23
		Total (%)	2 (2)	15 (14)	19 (17)	4 (4)	20 (18)	49 (45)	109	

SDQ = Strengths and Difficulties Questionnaire.

Per disorder (combination), cells containing numbers for content-wise matching SDQ profiles are printed in the lightest shade of grey, cells containing numbers for a possible/probable SDQ scoring algorithm prediction are shaded in the middle shade of grey, cell containing percentages for both a content-wise matching SDQ profile and a possible/probable SDQ scoring algorithm prediction are shaded in the darkest shade of grey.

[a] Numbers based on the modal cluster outcomes
